# Energy-Efficient and Highly Reliable Geographic Routing Based on Link Detection and Node Collaborative Scheduling in WSN

**DOI:** 10.3390/s24113263

**Published:** 2024-05-21

**Authors:** Minghua Wang, Ziyan Zhu, Yan Wang, Shujing Xie

**Affiliations:** School of Electrical Engineering, University of South China, Hengyang 421001, China; mhwang@usc.edu.cn (M.W.); zzysiyecao@163.com (Z.Z.); shujingx@usc.edu.cn (S.X.)

**Keywords:** geographic routing, link detection and repair, node collaborative scheduling, energy optimization

## Abstract

Energy efficiency and data reliability are important indicators to measure network performance in wireless sensor networks. In existing research schemes of routing protocols, the impact of node coverage on the network is often ignored, and the possibility that multiple sensor nodes may sense the same spatial point is not taken into account, which results in a waste of network resources, especially in large-scale networks. Apart from that, the blindness of geographic routing in data transmission has been troubling researchers, which means that the nodes are unable to determine the validity of data transmission. In order to solve the above problems, this paper innovatively combines the routing protocol with the coverage control technique and proposes the node collaborative scheduling algorithm, which fully considers the correlation characteristics between sensor nodes to reduce the number of active working nodes and the number of packets generated, to further reduce energy consumption and network delay and improve packet delivery rate. In order to solve the problem of unreliability of geographic routing, a highly reliable link detection and repair scheme is proposed to check the communication link status and repair the invalid link, which can greatly improve the packet delivery rate and throughput of the network, and has good robustness. A large number of experiments demonstrate the effectiveness and superiority of our proposed scheme and algorithm.

## 1. Introduction

Internet of Things (IoT), which is widely used in real life and brings great convenience to people’s lives, is a really hot research topic. The operation of IoT cannot be separated from the sensing and transmission of data. The wireless sensor network (WSN), as an important tool for data collection and forwarding in IoT, has a high research value [1].

The sensor nodes in WSN are small in size, easy to deploy, low in energy consumption, independent of each other, and usually deployed in the field of some specific monitoring areas. WSN is responsible for data sensing and transmission, mainly including sensor nodes and sink, sensor nodes sense the data and then transmit the data to the sink through one hop or multi-hop. WSN is widely used in the field of natural disaster prevention [2], agricultural and animal husbandry environment monitoring [3], and health monitoring because of its many advantages [4].

In practical applications, WSN has high requirements on the lifetime and reliability of sensor nodes, because WSN is usually randomly deployed in areas with harsh environments that jeopardize human safety or are difficult for humans to set foot in. When the batteries of sensor nodes are exhausted or network communication is interrupted, it will be more difficult and costly to manage and maintain the network manually, so it is necessary to design an energy-efficient and highly reliable routing protocol. Standard geographic routing is a routing protocol with low latency, few hops, and low energy consumption, which has become a hot research topic for many scholars in recent years [5]. However, this kind of routing also has the disadvantage of energy imbalance and unreliable data transmission. In the standard geographic routing protocol, the sensor nodes closer to the sink not only need to send the data they sense but are also responsible for forwarding the data sent by other nodes. In addition, it may face the problem of an infinite loop, which occurs when the sensor node is closer to the sink than its neighboring nodes, and it will result in the packets not being properly transmitted back to the sink.

The network energy imbalance creates a hotspot problem, which leads to network holes causing the whole network performance to go down [6]. An effective way to solve the energy imbalance in the network is to use the mobile sink [7], mobile sink can realize the energy balance of the network by changing the hotspot nodes around it through moving periodically. However, after using the mobile sink, the nodes in the network need to keep updating the new position of the sink for proper data transmission; flooding to notify all sensor nodes is not energy-efficient. Moreover, the mobile sink may create long paths and its movement causes some nodes in the network to be farther away from it, which increases the delay. Therefore, there is a need to use an efficient mobile sink strategy so that the energy consumed for network updates as well as the transmission delay is kept at a small level.

When facing the network unreliability problem, the standard geographic routing selects the nearest neighboring node to the sink as the next hop, but in the face of a multi-hop network environment, this strategy cannot guarantee that the node is able to accurately transmit data to the sink. When the data transmission link is broken or an infinite loop occurs, the sensor nodes continue to sense and transmit data, which undoubtedly consume unnecessary energy.

Most importantly, traditional routing protocols and transmission schemes always work with all the randomly deployed sensor nodes, which are required to sense, send and forward data. In a large-scale sensor network, the number of nodes deployed is hundreds or thousands, and the sensor nodes have a certain sensing range, such a huge deployment of sensor nodes will lead to the repetition of a large amount of redundant data, which may lead to the emergence of multiple sensor nodes monitoring the same points [8]. At this time, it is necessary to rationally schedule the sensor nodes to work, rather than allowing all the nodes to work, which can greatly reduce the energy consumption of the network. As a consequence, it is usually necessary to consider the partial coverage problem; by slightly reducing the quality of coverage in exchange for the network’s ultra-long lifetime, we obtain a higher packet delivery rate (PDR) and less delay because, for some applications such as fire monitoring and mudflow monitoring, these applications’ coverage rate can be changed based on the actual project requirement, so as to save energy.

In this paper, we divide the monitoring area into inner and outer areas, where the sink moves in the inner area and uses a tree-based routing protocol for network updating to save energy, and the outer area uses an improved geographic routing protocol, which adequately equalizes the energy consumption of the network. Based on this, we make the following contributions.

- We propose a link detection and repair scheme where each node periodically detects the state of its own transmission link and selects a valid neighboring node with higher energy as the next hop when the link is invalid, and when the node fails to find a valid link, the node stops the data transmission in order to conserve the energy, which greatly improves the reliability of the data transmission and saves energy to some extent.
- We combine routing protocol and coverage control technique to propose a node collaborative scheduling algorithm, which greatly reduces the number of packets generated and transmitted, largely conserves the energy of the network, and reduces the delay.

This paper is organized as follows: We will present some of the previous research works and analyze the advantages and disadvantages of these works in Section 2. In Section 3, the network model used for the study and the technical details and flows of the proposed algorithms will be described. In Section 4, the experimental methodology and parameters are provided, and the experimental simulation results are shown in terms of several important network metrics, and the simulation results are analyzed in detail. Finally, the work conducted in this paper and its value is summarized in Section 5.

## 2. Related Works

In recent years, in order to design energy-efficient routing protocols, researchers have combined various techniques such as clustering algorithms and machine learning algorithms, using ring routing structures, employing grid structures, and using mobile sinks. These methods have a large number of contributions to improve the network lifetime, but sometimes it is inevitable to sacrifice some network metrics such as packet delivery rate, end-to-end delay, throughput or network overhead. Therefore, when improving the network lifetime, we should not neglect the improvement of other network metrics so that the network has a strong comprehensive working capability and can adapt to the data collection in the case of no maintenance and management.

Routing protocols based on the clustering approach usually assume that the sensor nodes can send data directly to the sink instead of using multi-hop data transmission. In this approach, the cluster head in each cluster needs to collect the data from the member nodes and send them to the sink. In [9], the authors propose a clustering and routing algorithm for fast-changing (FC-CRAs) large-scale wireless sensor networks. In FC-CRAs, clusters are constructed based on the cluster radius, which dynamically adapts to changes in node energy and distribution. The FC-CRAs algorithm improves nodes’ survival time, increases network throughput, and saves network energy. In [10], the authors propose a novel distributed two-hop cluster routing protocol, where each sensor node obtains information about neighboring nodes within a two-hop range, the two-hop cluster is formed in a completely distributed way and the total transmission distance of inter-cluster communication is reduced. In [11], the authors exhibit an energy-saving clustering protocol based on adaptive Voronoi dividing, which effectively balances the energy consumption between cluster heads and cluster member nodes. The major drawbacks of these protocols are imbalanced energy consumption due to the frequent communication of the cluster heads and difficulty in practical applications in real life because of the limited single-hop communication range of sensors.

In recent years, machine learning-based routing protocols have been widely used in WSNs. Standard geographic routing only considers the distance to the sink of neighboring nodes to select the next hop and does not make more favorable decisions based on the status information of the surrounding nodes; such a path selection scheme is often not optimal at present. Literature [12] combines deep learning to study an efficient packet routing strategy, each node can learn to develop a good routing strategy, which effectively reduces the network’s energy consumption and improves the data transmission efficiency. In [13], the authors propose a multi-sink tactical mobile sensor network routing solution based on distributed multi-intelligent deep reinforcement learning (MADRL), which outperforms benchmark algorithms in terms of packet delivery rate (PDR), packet delivery time and energy efficiency. However, these machine learning algorithms need to obtain the status information of the surrounding nodes many times, which also increases the network overhead at the same time. In [14], the authors design a routing protocol with an inverse reinforcement learning concept in order to solve the problem of high network overhead caused by machine learning-based routing and the transmission environment of changing information due to node mobility; the network overhead and delay are reduced to some extent. However, the algorithm does not sufficiently reduce the energy consumption of the network.

Saving energy consumed by sensor nodes in the network has always been a very important challenge in WSN, and the ring routing structure has been proven to be effective in saving energy. In [15,16] introduce this routing structure, where nodes use geographic routing to send packets to the nearest ring node and obtain the location of the sink; however, this ring structure may introduce high latency. To solve this problem, in [17], the authors create multiple rings and update the sink’s location only to the nodes on the rings, thus reducing the network latency. Furthermore, in [18], the authors propose novel harvested energy scavenging and transfer capabilities in an opportunistic ring routing protocol, which comprehensively enhances the performance of the network and improves the high latency defects of ring routing; however, due to the high traffic around the ring routing structure, nodes near the ring are more likely to die and the energy consumption of the network is not balanced enough.

The grid structure is an energy-efficient network structure studied by scholars in recent years. In [19], researchers propose a routing protocol based on mode-switched networks, where a node is selected as a grid head in a divided grid, and the source node’s data transmission is accomplished by forwarding through the grid head, which improves the network lifetime to a great extent. Literature [20] comes up with a routing protocol based on a virtual grid structure, which uses the network structure to select specific nodes to save the location of the sink and share the information with common nodes, effectively reducing the network energy consumption and delay. In [21], the authors put forward a novel heuristic trust scheme that greatly improves the security and communication quality of the network, but this approach does not support mobile sinks.

It is necessary to make the sink move, a mobile sink can alleviate the hotspot problem to some extent and prolong the network lifetime. The use of a tree-based data dissemination model for mobile sinks greatly improves the network lifetime and reduces the network energy consumption in [22,23], but some nodes will go through longer paths for transmitting data back to the sink, which may lead to higher latency. In [24], the authors develop an energy-efficient routing protocol for large-scale I-IoT networks in a cloud-based SDN system and plan the optimal mobility paths for mobile sinks which effectively reduces the network energy consumption and latency, but with a low packet delivery rate. In [25], in order to solve the problems of unreliable data transmission (data transmission is trapped in an infinite loop) and routing hotspots in a standard geographic routing protocol, the authors put forward routing based on tree and geographic protocol (RTG), the algorithm divides the network into outer and inner areas, and the two areas use the improved geographic routing protocol and tree-based routing protocol, respectively, which balances the energy consumption of the network, and greatly improves the network lifetime, throughput, and delay, but the random mobility of the sink may lead to poorer network performance. The autonomous mobile sink based on the geographic routing protocol (AMSG) [26] further improves the network lifetime and throughput by allowing the sink to move autonomously based on RTG; however, due to the blindness of the geographic routing protocol, its packet delivery rate is not high.

Apart from data transmission in routing protocols, the coverage problem of WSN has also been a hot research topic. In [27,28,29,30] machine learning as well as approximation algorithms for optimization of disk-based coverage control are combined; the coverage of the network is improved and the network resources are fully utilized. However, the sensor nodes are required to be able to move, which increases the cost and is not applicable to large-scale networks. In addition, these studies are conducted on the traditional disk coverage, when the sensing point is outside the disk coverage, such data often cannot be collected; they do not take into account the collaborative work between the nodes. Finally, these coverage algorithms do not consider data connectivity.

Table 1 shows a summary of the advantages and disadvantages of the previous research work, these routing protocols use different methods to improve the performance of the network, but there are some shortcomings, one of the similarities is that none of these works take into account the impact of the coverage of the nodes, but use all the sensor nodes for the work although the routing protocols are designed to be really efficient. This does not make full use of the network’s resources, and the node’s energy may be wasted. In large-scale sensor networks, sensor nodes are usually deployed in large numbers in the network, which makes it likely that multiple sensor nodes cover the same spatial point, and if these redundant; repetitive data are transmitted back to the sink through multiple hops, not only causing a waste of information and network congestion but also accelerating the energy consumption of the network. Therefore, we need to determine which sensors are relatively independent of each other before the data transmission and select the least sensor nodes that meet our coverage expectation to work so that the reasonable allocation of network resources can largely extend the network lifetime and achieve high energy efficiency. This is also the core work of this paper. In addition, the standard geographic routing protocol has a certain blindness; data transmission can fall into an infinite loop, this problem has not yet been properly solved by the researchers, which is also one of the goals of this paper’s work. In this paper, we combine the geographic routing protocol with the node collaborative scheduling algorithm and link detection scheme and propose a scheme with a greatly comprehensive performance, which has high application value.

## 3. Proposed Method

In the standard geographic routing protocol, the sensor nodes are able to obtain their own geographic location and store the location of the sink. Each sensor node calculates the distance according to its location and the location of the sink and then selects the nearest neighboring node to the sink as the next hop. The minimum number of node hops is found by such a routing protocol, which results in lower latency and energy consumption, and it does not require flooding to obtain a path, making it an efficient, simple and scalable routing protocol [31].

**Figure 1:**
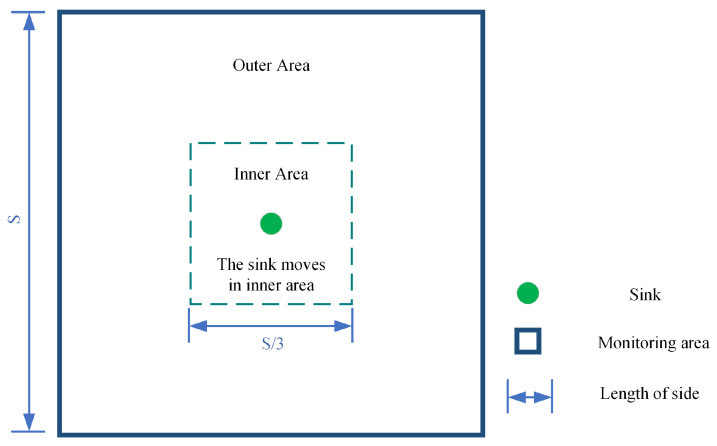
The network division of ERG.

In this paper, we first divide the monitoring area of size S×S into two parts, the inner area and the outer area, the inner area is of size $\frac{S}{3}\times \frac{S}{3}$, which is the center area of the monitoring area and the sink is allowed to move only within this range, and the outer area is the residual part of the monitoring area after removing the inner area, and this division of the area is proved to be suitable by the researchers [25]. Figure 1 shows the effect of the monitoring area after the division. The outer area and inner area use different routing protocols, the outer area uses the improved geographic routing protocol [25], while the inner area uses the tree-based routing protocol [25], which fully balances the energy of the outer area and inner area. Based on this, we propose energy-efficient and highly reliable routing (ERG) based on geographic location, which possesses a link detection and repair scheme that largely increases the packet delivery rate and throughput. Finally, we combine routing protocol with coverage control technique to propose energy-efficient and highly reliable geographic routing based on node collaborative scheduling (ERG-NCS), which greatly improves the coverage rate and network lifetime, further increases the packet delivery rate and reduces the network latency compared to other routing algorithms based on disk-based greedy scheduling algorithm (DGS).

### 3.1. Improved Geographic Routing Protocol

**Figure 2:**
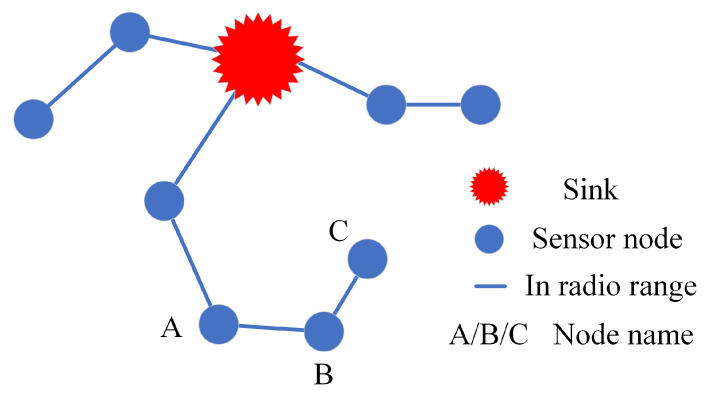
The advantage of improved geographic routing.

The outer area uses an improved geographic routing protocol which increases the reliability of the network compared to the standard geographic routing. Geographic routing has a drawback in that it may cause infinite loops of packets, which happens when all its neighboring nodes are farther away than it is from the sink; Figure 2 shows the schematic diagram of a node caught in an infinite loop. According to the standard geographic routing protocol, when node B generates a packet, since node C is closer to the sink than node A, node B transmits data to node C, and node C will only transmit data to node B. In this way, the data of node B and node C are trapped in an infinite loop. For this reason, the improved geographic routing defines a hopeful node, when a node exists at least one neighboring node closer than it is to the sink, such a node is called a hopeful node, and if it does not exist, such a node is a hopeless node, according to Figure 2, node A and node B are hopeful nodes and C is a hopeless node, so in the improved geographic routing protocol the node B will choose node A as the next hop, so the infinite loop is avoided. Secondly, to maintain the energy balance in the outer area, we will select the node that satisfies the energy threshold as the next hop. The initial energy threshold of each node is half of the initial energy, the node in the protocol prioritizes the nearest hopeful node that meets the energy threshold as the next hop, if there is no such a hopeful node, this energy threshold will be multiplied by $\frac{1}{2}$, and then continue to search for the nearest hopeful node as the next hop. When the energy threshold is less than the energy of sending the data once, the node will choose the next hop among the hopeless nodes. This improves the data reliability in the outer area and also balances the energy consumption. The pseudocode for the improved geographic routing is displayed inside Algorithm 1.
**Algorithm 1** Improved geographic routing algorithm**Input:** Neighboring nodes’ locations**Output:** NexthopEth ← the energy of sending data onceThe node judges its distance to sink compared with its neighbor nodes**if** (there is at least one node closer to sink than it) **then**  Node.hopeful ← True                     //It is a hope node**else**  Node.hopeful ← False                   //It is not a hope node**end if**Define choosing mode ID ← True   //True means selecting nexthop from hopeful nodesγ ← the initial energy of sensor nodesThe initial status of output Nexthop ← False    //False means no next hop is selectedMindis ← +∞                  //A big enough number for initialization$\gamma \leftarrow \frac{1}{2}\times \gamma$                       // Define an energy threshold**for** each neighbor node  **if** (Node.hopeful × ID == True) **and** (Node.energy > γ) **and**   (Mindis > (the distance between neighbor node and sink)) **then**    Mindis ← the distance between neighbor node and sink    Nexthop ← neighbor node  **end if****end for****if** (Nexthop == False) **and** (γ > Eth) **then**  **goto** the distance between neighbor node and sink**else if** (Nexthop == False) **and** (γ < Eth) **then**  ID ← false                 // Now choose the nexthop from hopeless nodes  **goto** γ ← the initial energy of sensor nodes**end if****Output** ← Nexthop

### 3.2. Tree-Based Routing Protocol

**Figure 3:**
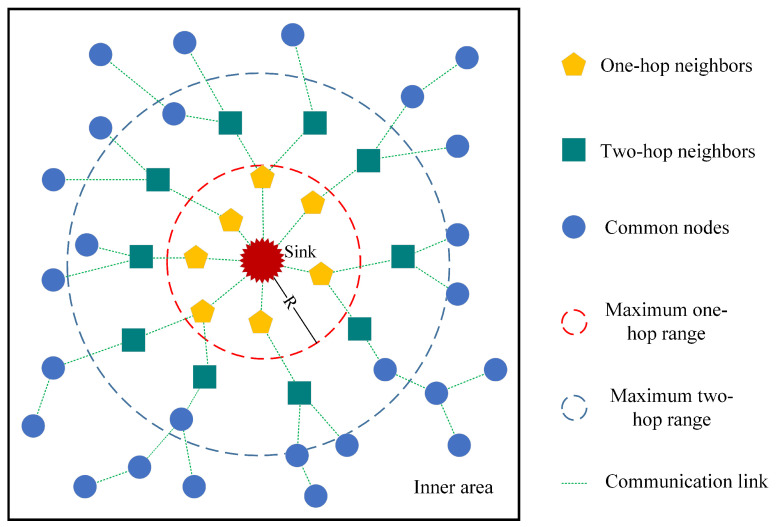
The schematic of the tree-based routing protocol.

In WSN multi-hop routing, when the sink is in a static state, the nodes closer to the sink have high traffic, they are not only responsible for sending their own data but also act as relay nodes to forward the data sent by the more distant nodes, they are more likely to die due to energy depletion, so it is essential to allow the sink to move. The mobile sink is able to constantly change the neighboring nodes around them to balance the energy consumption of the network, whereas the sink is allowed to move only in the inner area, which reduces the latency and energy consumption. At the very beginning, all the nodes store the initial position of the sink, which is located right in the center of the entire monitoring area, and the nodes in the inner area are connected to the sink through the geographic routing protocol. The sink only needs to notify the one-hop nodes and the two-hop nodes of its new position after each move instead of notifying the whole network through flooding, which is definitely energy-efficient. Figure 3 reveals the schematic diagram of the tree-based routing protocol, the yellow nodes represent the one-hop neighbor nodes of the sink, the green nodes represent the two-hop nodes of the sink, the blue nodes are the normal nodes in the inner area, and *R* represents the communication radius of the sink. The sink moves in the inner area and sends a “my new location” control packet to its one-hop neighbor nodes for every *R* distance moved, and the one-hop neighbor nodes will continue to send a “sink new location” control packet to their nearest neighbor nodes after receiving this command, which is called two-hop nodes, and all other nodes in the network can connect to sink through these nodes. when the sink moves, the original one-hop node will select the nearest neighbor node of the original sink as the next hop if it is out of the radio range of the current sink. Each time a new location of the sink is stationed in the network for a while to collect the data of the whole network, the sink has to maintain the periodical movement to balance the network energy consumption in the inner area.

### 3.3. Link Detection and Repair Strategy

**Figure 4:**
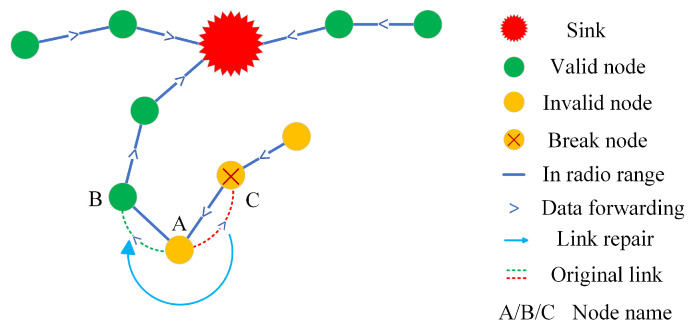
The schematic of link detection and repair process.

The improved geographic routing protocol and tree-based routing protocol fully balance the network energy consumption and prolong the network lifetime. However, in some communication environments with more complex node distribution, the reliability of the network cannot be fully guaranteed. Figure 4 demonstrates this situation, when node A transmits data, nodes B and C are both hopeful nodes, but node A will choose node C as the next hop because it is closer to sink, which leads to an infinite loop. It can be seen that selecting the hopeful node as the next hop does not ensure that the data can be transmitted back to the sink successfully.

In this regard, we propose a novel link detection and repair scheme, we propose valid node, invalid node, and break node in this scheme based on the concepts of hopeful node and hopeless node. We define a valid node as a node whose data can be transmitted back to the sink successfully through single hop or multiple hops, while the data of a node that cannot be transmitted back to the sink is an invalid node, and a break node is a node whose next hop is about to fall into an infinite loop or a node whose next hop is missing. This link detection and repair scheme has three stages.

- First stage: Link detection

Each node periodically sends a link detection command, which is transmitted along only a selected next hop and does not require a flooding approach. At the beginning, all nodes are defaulted as valid nodes, and the node detects along the next hop, once the break node is detected, the break node will send a feedback message to go back along the original route and mark all the nodes on the route as invalid nodes. The node whose data arrive at the sink normally does not need a feedback message, marking it as a valid node by default, so that the detection is energy-efficient.

- Second stage: Link repair

After nodes are marked as invalid nodes, each invalid node sends a “is it a valid node” command to the neighboring nodes, and if there are valid nodes in the neighboring nodes, they will send residual energy information to the invalid node, the invalid node picks the node with the largest remaining energy as the next hop based on the remaining energy information it receives and marks the status of the invalid node as a valid node. The invalid nodes that have not found a valid neighbor node go to the third stage.

- Third stage: Link examination

All invalid nodes send inquiry commands along the next hop, and the node that receives the inquiry command starts to judge if it is a valid node, then it replies the “node is valid” message to the corresponding node along the previous hops. If it is not a valid node, it continues to send the inquiry command to the next hop. If a valid node appears in the path to the original break node, the node continues to transmit data and marks its status as a valid node. On the contrary, the invalid node enters into energy-saving mode and stops data transmission to conserve energy and waits for the next path detection.

Algorithm 2 shows the pseudocode of the link detection and repair process. This link detection and repair scheme effectively solves the blindness as well as unreliability problem of the data transmission in the improved geographic routing protocol, which is a further optimization and modification of the improved geographic routing protocol, and greatly improves the inaccuracy problem of data transmission due to the complex distribution of nodes as well as the routing hole problem that occurs with the death of nodes. In Figure 4, after the data sent by node A encounter break node C, the link repair strategy is enabled and successfully connects to the valid node B, and the network resumes normal communication. In addition, to ensure the rationality of the network, we consider the network paralyzed when the sink has a very small periodical packet collection rate several times in a row, the node exits the energy-saving mode and dies normally.
**Algorithm 2** Link detection and repair algorithm**Input:** Sensor nodes’ next hop before repairing**Output:** Sensor nodes’ next hop after repairingNode ← each sensor node**if** (meet the break node while data forwarding) **then**         // Start link detection  Node.status ← False                  // False means invalid node  **for** each neighbor node S               // Traverse each neighbor node   **if** (S.status == True) **then**                     // Start link repair      Ecol ← each neighbor node’s remaining energy   **end if**  **end for**  **if** (Ecol is not empty) **then**         //It exists valid nodes in its neighbor nodes     Lic ← selects the node with the biggest remaining energy as the nexthop in Ecol     Node.nexthop ← Lic     Node.status ← True  **end if****end if****if** (Node.status == False) **then**                 // Start link examination    Exa ← the link examination result  **if** (Exa == True) **then**                   // True means link is valid     Node.status ← True                 // Still choose the initial next hop  **else**     Node.nexthop ← False     The node stops data forwarding  **end if****end if****Output** ← Node.nexthop

### 3.4. Disk-Based Greedy Scheduling Algorithm (DGS)

As mentioned earlier, since sensors have a certain coverage area, and inside the classical routing algorithms, they use all the nodes for data transmission, which is obviously very energy-consuming, and will also bring a large network load. Therefore, we can set a network coverage rate threshold, which is usually given by the actual engineering, and reasonably schedule the nodes that satisfy the coverage rate threshold to work efficiently. In the classical coverage problem study, the coverage model of sensor nodes is considered as disk coverage. The disk coverage model can be expressed as Equation (1).

(1) $$f\left(d\left(n,p\right)\right)=\left\{\begin{array}{c} 1\\ 0 \end{array}\;\begin{array}{c} if \end{array}\right.\;\begin{array}{c} d\left(n,p\right)\leq R_{s}\\ otherwise \end{array}$$

where $d\left(n,p\right)$ is the distance from the sensor node *n* to the spatial point *p*, and $R_{s}$ is the sensing radius of the sensor node. It can be seen from the formula when the spatial point *p* is located within the sensing radius of the sensor node, the data of the spatial point can be sensed, but beyond this range, it cannot.

At the very beginning, all the sensor nodes have to send their current geographic locations to the sink, while the sink stores the sensing radius of the sensor nodes and the coverage rate threshold. After the sink receives the nodes’ locations, it selects the least sensor nodes required to reach the coverage rate threshold for data collection according to the DGS, and some of the rest of the nodes may have to participate in the forwarding of the data, and the remaining nodes are dormant. The core idea of DGS is to use sensor nodes as little as possible to achieve the corresponding coverage rate requirement. DGS selects a sensor node with the largest coverage each time through multiple iterations until the coverage rate threshold is reached. Whenever a node dies, the sink will re-schedule the nodes to work or sleep.

**Figure 5:**
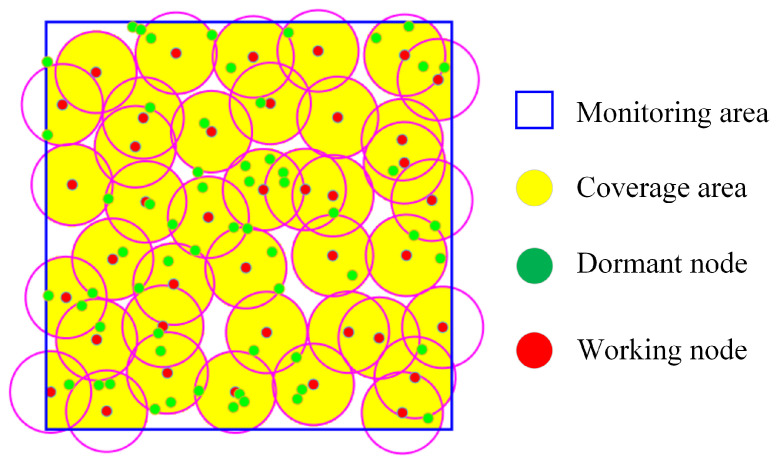
The schematic of the DGS protocol.

Figure 5 exhibits a schematic of DGS, where 100 sensor nodes are randomly deployed in the monitoring area at a coverage rate threshold of 0.9, and the sink obtains the location of each sensor node and can manage them to work. The yellow area is the area covered by the scheduled nodes, the nodes marked in red are working nodes. Some of the green nodes may become relay nodes for data forwarding, and the others are in the dormant state, this management makes the network operate efficiently.

### 3.5. Node Collaborative Scheduling Algorithm (NCS)

Although the DGS algorithm can largely exploit the advantages of the disk coverage model, the disk coverage model also possesses certain limitations and such data are not captured when the sensed points are outside the disk. Therefore, we use a node collaboration algorithm [32] to reconstruct the points that are not sensed by the sensor nodes by deeply excavating the spatial correlation between the nodes, which greatly increases the coverage of the network. Apart from that, we further propose a node collaborative scheduling algorithm with the same coverage rate threshold, fewer nodes can be scheduled to work. The node collaboration coverage model function expression is shown in (2).

(2) $$\xi \left(p\right)=\sqrt{\frac{1}{T}\times \sum_{t=1}^{T}{(\left(u^{t}\left(p\right)-{\hat{u}}^{t}\left(p\right)\right)}^{2})}$$

ξ(p) is the time-averaged root mean square error (RMSE) of the reconstructed spatial point *p*, and $u^{t}\left(p\right)$ and ${\hat{u}}^{t}\left(p\right)$ denote the perceived true and estimated values of the spatial point *p*, respectively. We define that a spatial point *p* is considered to be a valid reconstructed sensing point when the RMSE of the reconstructed point *p* is lower than a set threshold λ. λ is usually given by the demand of the actual project and represents the reconstructed quality of the spatial point *p*.

**Figure 6:**
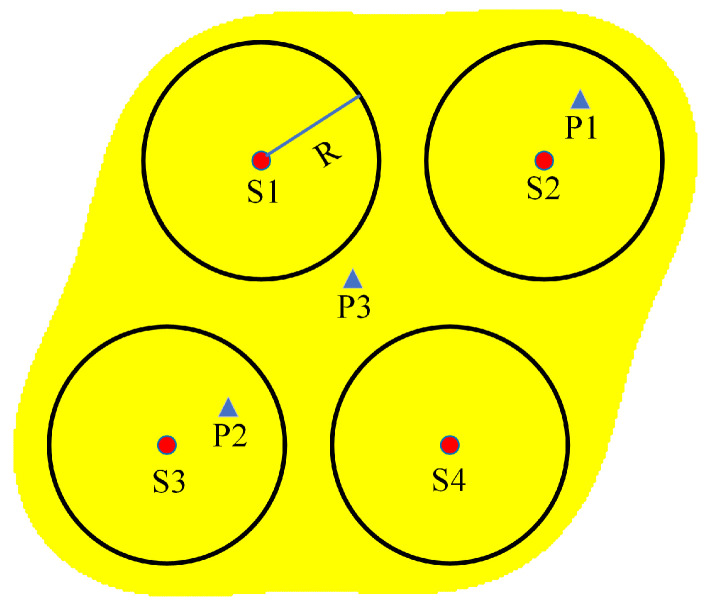
A schematic of four nodes collaboration.

Figure 6 shows an example of a node collaboration coverage model. S1, S2, S3, S4 are the sensor nodes, the green circle represents the coverage range of the disk model, *R* is the sensing radius of the sensor nodes, P1, P2, P3 are the three spatial points, and the yellow area is the coverage of the nodes after collaborative coverage. Obviously, the disk coverage model can only sense P1 and P2 located within the sensing radius and it does not have the ability to sense P3, which can be sensed by the node collaboration coverage model.

We divide the monitoring area of S×S into several reconstructed subregions with edge length *a*. The whole reconstructed subregion is within the range of the variable range. When two or more nodes are located within the range of the variable range, the nodes can collaborate with each other, while when the distance between the nodes exceeds the range of the variable range, it is considered that there is no spatial correlation between the nodes to collaborate with each other. In the reconstructed subregion, five reference points are set. Equations (3) and (4) reflect the coordinates of these five reference points.

(3) $$HC=\left\{0.2a,0.8a,0.5a,0.2a,0.8a\right\}$$

(4) $$VC=\left\{0.2a,0.2a,0.5a,0.8a,0.8a\right\}$$

HC is the horizontal coordinates of the five reference points and VC is the vertical coordinates of the five reference points. In a reconstructed subregion, five nodes nearest to the corresponding reference points are scheduled to work together, and these five reference points are chosen because when the sensor nodes’ positions are completely overlapped with these five reference points, the collaborative coverage can completely cover the whole reconstructed subregion, and the coverage range is the biggest at this time. In addition, when the nodes are unevenly distributed, the nodes scheduled to work together can also have a high coverage rate. The network always ensures that each reconstructed subregion can reach the coverage rate threshold, when the reconstructed subregion fails to reach the coverage rate threshold, nodes closer to the center of the reconstructed subregion will be gradually added to collaborate until the subregion coverage rate threshold is met, and when the coverage of the whole network fails to reach the coverage rate threshold, all remaining nodes will be mobilized to carry out the work.

The ERG-NCS algorithm is shown in Algorithm 3. In the beginning, all sensors are divided into inner or outer areas according to their geographic locations, and they need to confirm which reconstructed subregion they belong to, then all sensor nodes send their current geographic locations to the sink, and the sink selects the nodes that satisfy the coverage rate threshold in each reconstructed subregion according to the NCS to collect data. When a node dies in the reconstructed subregion, the nodes that satisfy the coverage rate threshold will be re-scheduled to work. We call the process of all sensor nodes generating data once to the sink receiving the data as a round, the improved geographic routing protocol, tree-based routing protocol, and the link detection and repair protocol in the algorithm are not carried out in every round, they run frequently will cause a large amount of wasted network energy and accelerate the paralysis of the network, and run too few is difficult to obtain the ideal network performance, they have an optimal number of rounds running value.
**Algorithm 3** ERG-NCS algorithm**Input:** The network parameters**Output:** The network performanceNetwork initialization and partitioning    // To determine their neighbor nodes and positionsEach sensor node sends its location to sink**for** each reconstructed subregion                     // Start NCS process    **for** each node in this reconstructed subregion     Find the five nodes closest to five maximum coverage contribution points to collaborate    **end for**    The sink notifies these nodes to work for data collection**end for****for** each sensor node in outer area         // Start improved geographic routing process    Enter the algorithm 1 process**end for****for** each sensor node in inner area            // Start tree-based routing process    Sensor node selects nexthop according to geographic routing protocol    All the nodes can connect to the sink, which formulates the tree structure    Sink moves a communication distance and notifies its two-hop nodes    A new tree structure is created**end for****for** each working node               // Start link detection and repair process    Enter the algorithm 2 process**end for****while** (the network lifetime is not over)    Perform energy calculation and count communication rounds    **if** (a node is dead)    **goto** NCS process    **end if****end while**

**Figure 7:**
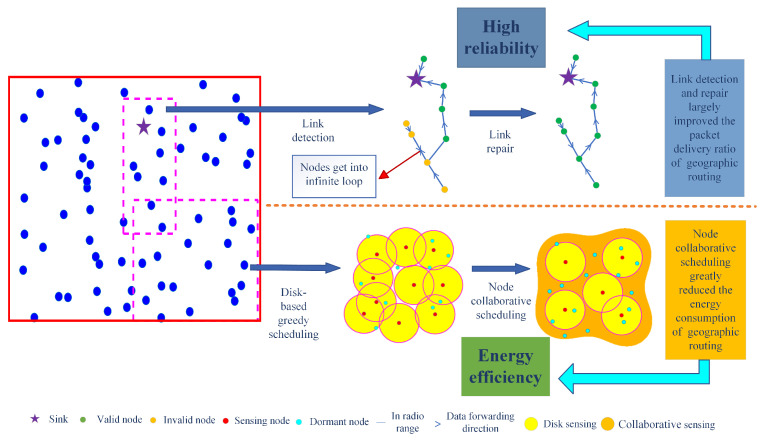
Link detection and repair and node collaborative scheduling principles.

Figure 7 demonstrates the principles of link detection and repair and node collaborative scheduling. The link detection and repair scheme allows nodes to clearly obtain their link status and select a reliable node as the next hop, which effectively solves the problem of data transmission caught in an infinite loop. The node collaborative scheduling algorithm makes full use of the spatial correlation between nodes and further reduces the number of working nodes on the basis of the disk-based greedy scheduling algorithm, which reflects the contribution of the proposed algorithm to improve the energy efficiency of the network as well as the reliability of the network.

## 4. Performance Evaluation

In this section, we give the energy model for conducting simulation experiments and the related simulation parameters and we define some important routing simulation metrics. Finally, when we conduct the experiments, we compare the proposed ERG-NCS with ERG-DGS, AMSG-DGS, RTG-DGS, ERG, AMSG, and RTG, and through the comprehensive analysis, the ERG-NCS is better than any of them.

### 4.1. Energy Calculation Model

In the energy calculation approach, we use the energy loss calculation model given in [32]. It includes formulas for calculating the energy loss for sending and receiving data and we will use these formulas in our simulation experiments, Equation (5) is the formula for calculating the energy consumed for sending and forwarding data.

(5) $$E_{Tx}\left(h,d\right)=\left\{\begin{array}{cc} hE_{elec}+h\varepsilon_{fs}d^{2} & d<d_{0}\\ hE_{elec}+h\varepsilon_{mp}d^{4} & d\geq d_{0} \end{array}\right.$$

where $E_{elec}$ represents the energy consumed to send one bit of data, and *h* is the number of bits occupied by the sensed data packet. $\varepsilon_{fs}$ and $\varepsilon_{mp}$ represent the energy loss factor for data sending under the free space model and multi-path fading model, respectively, the calculation model needs to be specifically selected based on the distance between nodes. When the distance *d* between nodes is less than $d_{0}$, the free space model is selected. Otherwise, when *d* is greater than or equal to $d_{0}$, it is considered that the distance between nodes is far, and the multi-path fading model is used for data transmission. $d_{0}$ is calculated by Equation (6), and its value is a constant value.

(6) $$d_{0}=\sqrt{\frac{\varepsilon_{fs}}{\varepsilon_{mp}}}$$

The formula for the received energy calculation is represented by (7), which is equal to the product of the size of the received packet multiplied by $E_{elec}$, it is independent of the distance between the nodes.

(7) $$E_{Rx}\left(h,d\right)=hE_{elec}$$

### 4.2. Simulation Metrics and Parameters

Next, we define the performance metrics that appear in the simulation experiments. We simulate some core routing performance such as network lifetime, throughput, packet delivery rate, and end-to-end average delay. In addition, we add coverage rate to judge the advantage of the algorithm in network coverage, the following are some definitions of these evaluation metrics.

Network lifetime: defined as the number of rounds of packet transmission iterations when the percentage of dead nodes in the network reaches 5%, the coverage-related algorithms need to meet the coverage rate threshold. The reason why 5% is defined is because the network has high reliability and coverage rate at this time.

Throughput: defined as the total number of packets successfully received by the sink during the network lifetime.

Packet delivery ratio(PDR): defined as the ratio of the total number of packets successfully received by the sink to the total number of packets that the network should generate during the network lifetime. Equation (8) represents this calculation.

(8) $$Pdr=\frac{Rec}{Gen}$$

End-to-end average delay: defined as the ratio of the sum of the time incurred by all the nodes from the sending of the data to the reception of the data by the sink to the number of nodes. Equation (9) exhibits this calculation.

(9) $$D_{ave}=\frac{D_{all}}{Num}$$

Network coverage rate: defined as the ratio between the range covered by the working nodes and the monitoring area. Equation (10) shows this calculation.

(10) $$Cov=\frac{C_{w}}{S^{2}}$$

Coverage rate threshold: defined as the minimum network coverage rate required to achieve network operation, its value is usually given by actual engineering.

Table 2 gives the parameter settings of the simulation experiment, we use MATLAB 2018a as the simulation experiment platform, MATLAB contains the advantages of programming efficiency, perfect functions, and powerful calculation ability.

### 4.3. Simulation Result

We compare the proposed ERG with AMSG as well as RTG, both of which are excellent energy-efficient algorithms based on geographic routing and have significant advantages in extending the lifetime of multi-hop networks. After that, we combine ERG with the DGS algorithm and NCS algorithm to improve the performance of the network comprehensively, so the final algorithm we propose is ERG-NCS. The following are the experiments we did, the average value of the simulation results is taken for 10 runs.

The two main aspects of this paper are to improve the energy efficiency and data reliability of the network, the improvement of energy efficiency is reflected by the network lifetime and network energy consumption in the simulation results, the higher the network lifetime and the lower the energy consumption, the higher the energy efficiency of the network. As for the data reliability of the network, it is reflected by the packet delivery rate and the robustness of the network in the experiment; the higher the packet delivery rate and the better the network robustness, the more reliable the network is.

**Figure 8:**
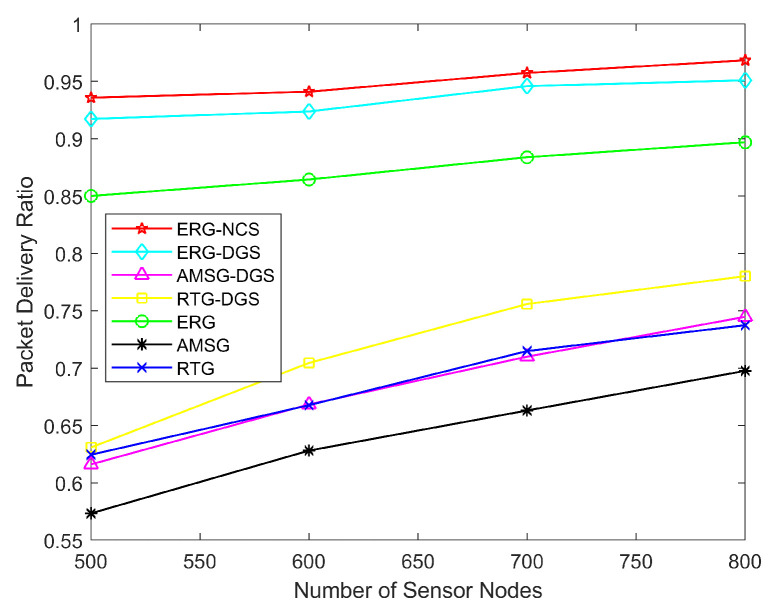
Network PDR with different number of sensor nodes.

Figure 8 exhibits the network PDR with a different number of sensor nodes. The ERG is able to detect and repair paths periodically, which greatly improves the reliability of the network, and thus the PDR of ERG is much higher than RTG and AMSG. On the other hand, RTG makes data collection less prone to produce holes due to the random mobility of nodes, and thus the PDR of RTG will be a bit higher than that of AMSG. Apart from that, the algorithms after considering the coverage are able to have more path selection options in data transmission because the nodes are less prone to die, thus the PDR of both NCS and DGS is a bit higher than the original algorithms, especially for NCS, as the nodes selected for work are the least number of nodes, the effect of such an increase is more obvious, and thus the PDR of ERG-NCS is the highest. Table 3 shows the PDR of these algorithms with different numbers of nodes, the underlined value is the highest PDR for each node number. The packet delivery rate of ERG is improved by 48.40% and 38.54% on average over the baseline algorithms AMSG and RTG, respectively, and this improvement is significant. After considering the node collaborative scheduling algorithms, the ERG-NCS algorithm is improved by 8.79% on average over the ERG, which is a good indication of the superiority of this network resource allocation.

**Figure 9:**
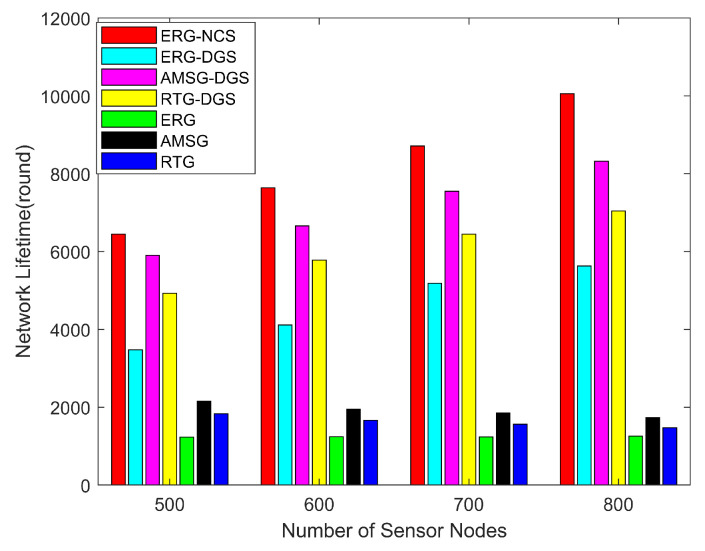
Lifetime with different number of sensor nodes.

Figure 9 shows the network lifetime with different numbers of sensor nodes. Generally, as the number of nodes increases, the packets of the network increase, the energy consumption is accelerated and the network lifetime decreases. However, the considered coverage algorithms make full use of the network resources and generate packets by selecting only the nodes that satisfy the coverage rate threshold, so the considered coverage algorithms’ lifetime is much larger than the original routing algorithms, and as the number of nodes increases the network’s ready-to-work nodes also increase, so the network lifetime becomes longer. Although ERG has excellent PDR, it also leads to faster death of nodes in the inner area, so the network lifetime of ERG is a little lower. The autonomous mobile sink of AMSG is able to extend the lifetime of nodes around the sink, so the lifetime is a little higher than RTG. In considering the coverage algorithm, the network lifetime of ERG-DGS becomes quite longer. On this basis, our further proposed NCS fully considers the spatial correlation among nodes, and thus is able to satisfy the coverage rate threshold with fewer monitoring nodes, so the ERG-NCS has the longest network lifetime, and simultaneously achieves a high PDR with a high network lifetime. Table 4 gives the network lifetime of these algorithms with different numbers of nodes, the underlined value is the highest lifetime for each node number. The network lifetime of ERG-NCS is improved by an average of 3.27 times over AMSG and 4.03 times over RTG, which is due to the high number of nodes in a large-scale sensor network, which makes this gain especially effective after scheduling nodes to work. In addition, the lifetime of ERG-NCS is also improved by an average of 15.57% and 35.79% over AMSG-DGS and RTG-DGS, which results from the high efficiency of the NCS algorithm.

**Figure 10:**
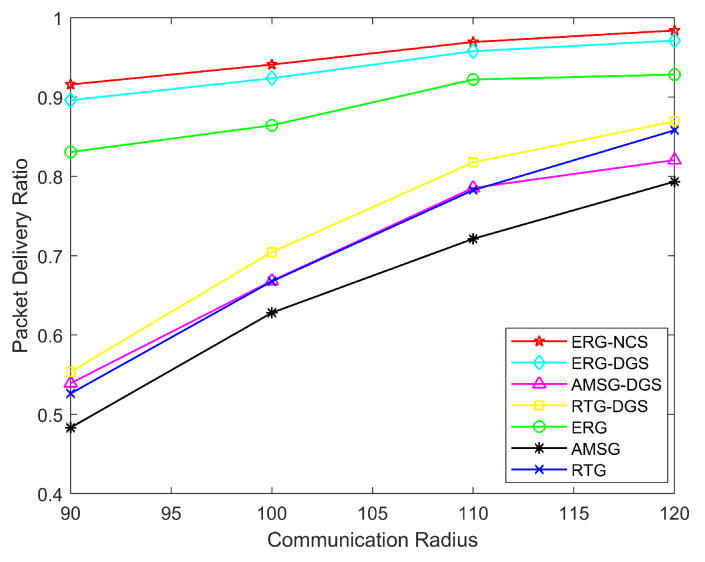
Network PDR with different communication radius.

Figure 10 demonstrates the network PDR with different communication radii. When the communication radius increases, the node has more next-hop nodes to choose from, and thus it has more paths to choose from, which betters the network communication environment, so the PDR increases with the communication radius. The ERG is able to repair the broken paths, and the PDR of ERG is not affected by communication radius as much as AMSG and RTG, and it can be said that ERG’s robustness is superior to that of the AMSG and RTG. In addition, the differences of these algorithms remain consistent with Figure 8.

**Figure 11:**
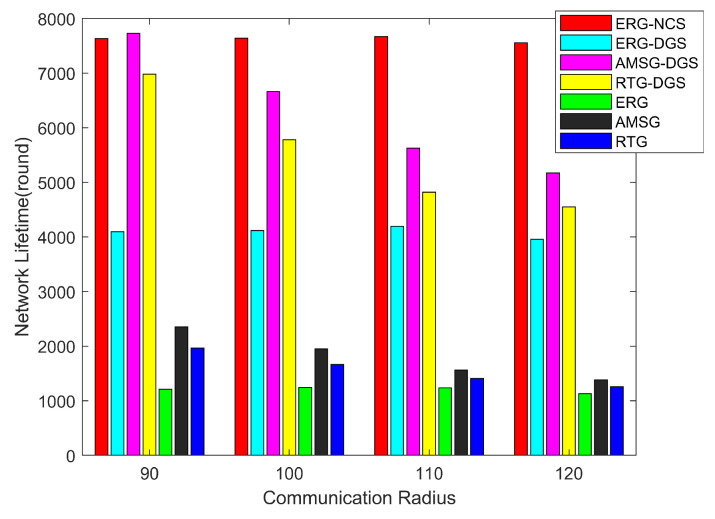
Lifetime with different communication radius.

Figure 11 measures the network lifetime with different communication radii. As mentioned earlier, as the communication radius increases, the communication environment of the entire network becomes better and packets are more likely to be transmitted back to the sink successfully through multiple hops, so the energy consumption of the RTG and AMSG networks increases with the increase in the communication radius, thus leading to a decrease in the network lifetime. For the ERG algorithm, when the network communication environment is not good, it also repairs the links and saves the energy of the broken links, thus it does not change significantly due to the change in the communication radius, which further demonstrates that ERG is very robust and ERG-NCS is able to maintain a high network lifetime all the time.

Generally, as the communication radius of sensor nodes increases, although the communication environment of the network becomes better, it aggravates the node energy consumption and the network lifetime will decrease, but ERG-NCS has better robustness, which means that it has a strong dynamic adjustment ability, and the network lifetime decreases not obviously, so in the figure it has an optimal communication radius of 120 m, and at this time, it has the highest packet delivery rate and the lowest network latency, and the network lifetime is almost unaffected.

Next, the variation in network energy consumption, number of alive nodes, network coverage, throughput, and PDR with the number of rounds is explored for the same nodes distribution scenario with the number of nodes being 600, communication radius being 100 m, and coverage rate threshold being 0.9.

**Figure 12:**
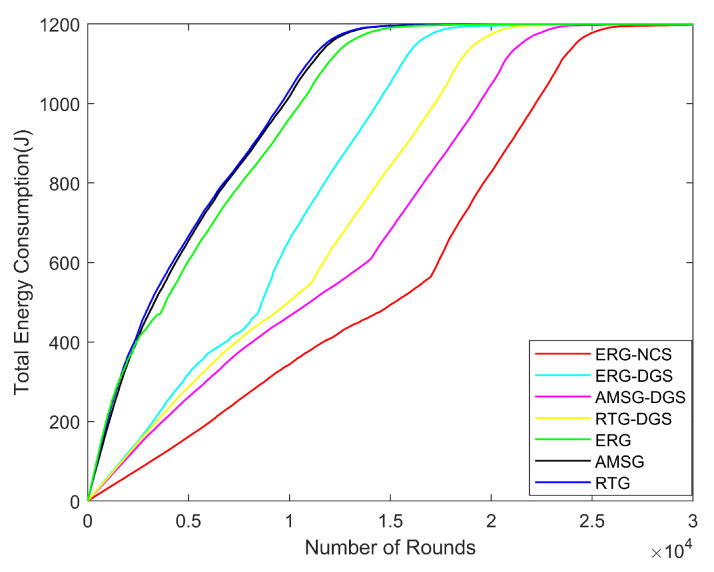
Total energy consumption with different number of rounds.

From Figure 12, it can be seen that due to the difference in the number of monitoring nodes selected by different algorithms, each monitoring node creates a path by single hop or multiple hops. The lesser the number of working nodes selected, the lesser the number of paths created, which saves the network energy considerably, so the energy consumption of ERG-NCS is minimized. At first, the energy consumption of ERG is higher than RTG and AMSG, but ERG is able to save the energy of broken links and when a certain number of rounds is reached, the energy consumption of the network is a little bit smaller, of course, this energy saving mechanism is not apparent after considering coverage. There is a turning point when the curve reaches a certain number of rounds in the considered coverage algorithms, this is because the networks are no longer able to meet the coverage rate threshold at this point, thus mobilizing all the nodes to work. The amount of energy consumption deeply affects the lifetime of the network, and the ERG-NCS has the lowest energy consumption so that it can continuously run for the maximum number of rounds.

**Figure 13:**
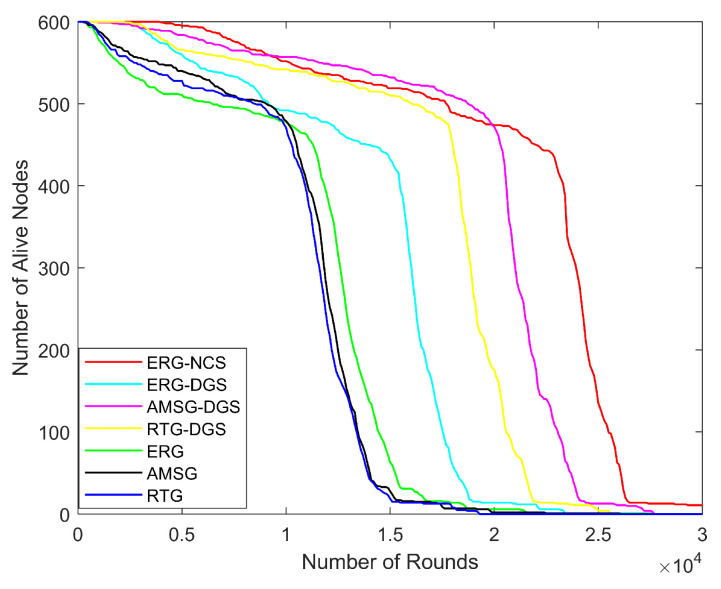
Alive nodes with different number of rounds.

In Figure 13, the number of alive nodes is closely related to the energy consumption of the network, when all the nodes are selected to work, the network energy consumption is high and the nodes die faster. ERG-NCS makes full use of spatial correlation among the nodes, which enables the coverage rate threshold to be reached with fewer sensing nodes, and thus the nodes’ death rate is the slowest. The sink in the AMSG is able to move to the area where the nodes are burdened heavily, thus effectively prolonging the nodes’ lifetime around the sink, so the lifetime of AMSG is a little longer than RTG. The nodes’ death is categorized into two trends, the first slower trend is the nodes that die in the inner area, which are close to the sink and are more likely to die due to energy depletion. The second trend is mainly caused by the nodes in the outer area, and the nodes in the outer area start to die centrally when they reach a critical point due to balanced energy consumption, from this aspect it can also be seen that the above algorithms especially ERG-NCS make full use of the nodes’ energy.

**Figure 14:**
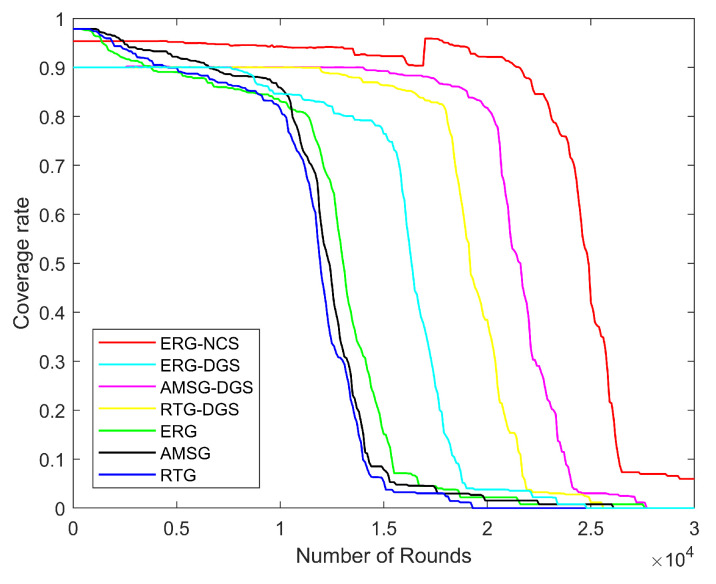
Coverage rate with different number of rounds.

Figure 14 illustrates the graph of network coverage rate with the number of rounds, and it can be clearly seen that with a coverage rate threshold of 0.9, ERG-NCS needs to sacrifice little coverage rate at the beginning in exchange for high coverage rate for the longest period of time, this is because in a network with a large number of sensor nodes, there is a large amount of redundancy and duplication of data, which will degrade the performance of the network, and scheduling algorithms can be a good way to avoid this risk. Allowing the coverage range to be relatively independent of the sensor nodes to work, especially after taking into account the collaboration between the nodes, the network can use fewer working nodes and also have a better coverage effect, which indicates that this nodes’ scheduling approach is very efficient. In contrast, RTG, AMSG, and ERG all use all nodes for their work which generate a large amount of redundant data. In addition, the initial coverage rate of ERG-NCS is also higher than that of the DGS, which gives the algorithm some room for error tolerance, further reflecting the superiority of the NCS method compared to the DGS method.

**Figure 15:**
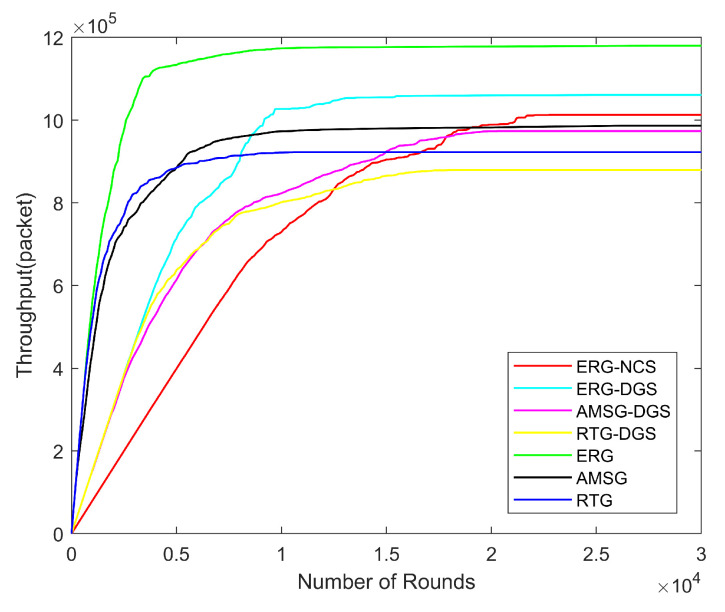
Throughput with different number of rounds.

Figure 15 reveals the network throughput with different numbers of rounds. ERG has the highest throughput due to its efficient energy management method and high PDR. After considering the coverage algorithms, the throughput of the networks decreases a bit compared to the original algorithms which is due to the fact that there are fewer sensing nodes after the nodes’ scheduling, which leads to a reduction in the number of packets generated. However, the throughput of the ERG-NCS is still higher than that of RTG and AMSG. In addition, the data collected by ERG-NCS are much less repetitive and redundant than that without considering the coverage algorithm, which means that the data collected by ERG-NCS are the most effective.

**Figure 16:**
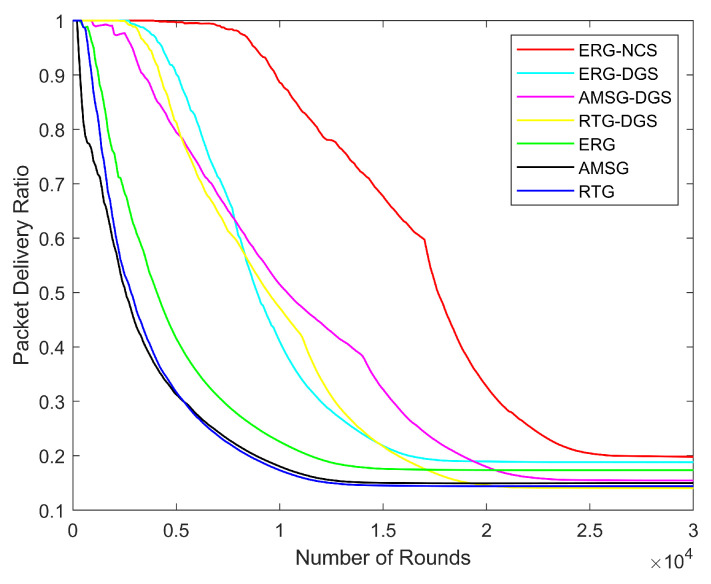
Network PDR with different number of rounds.

It is evident from Figure 16 that ERG’s link repair effect makes it much more reliable than RTG and AMSG, the nodes can determine the state of the communication link, and as long as a valid node is selected as the next hop, then the data can definitely be transferred back to the sink successfully. After scheduling the network nodes, having fewer monitoring nodes also results in more alternative paths for data transmission, allowing the network to maintain a high PDR for an extended period of time. Additionally, an excellent node collaboration model, nodes’ scheduling and link repair capabilities enable ERG-NCS to outperform others significantly in terms of PDR.

**Figure 17:**
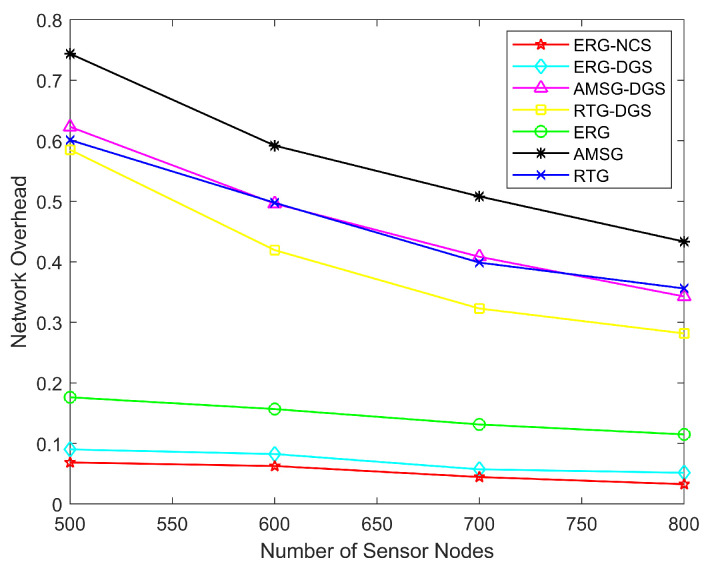
Network overhead with different number of nodes.

Figure 17 shows how the network overhead varies with the number of nodes. The ERG-NCS algorithm suffers from packet loss only if the link cannot be repaired, and in most cases, especially when the death number of nodes in the inner area is low, the data are usually transmitted back to the sink successfully. Therefore, the network overhead of ERG-NCS is the lowest, and it suffers from the smallest number of packet loss, while the sink receives the most data. For RTG and AMSG and their derived algorithms, the communication of the network cannot be guaranteed exactly because geographic routing possesses a certain degree of blindness, so their network overhead will be higher.

Next, we explore how the average end-to-end delay of ERG varies with the communication radius for the three cases of using the no scheduling algorithm using the disk-based greedy scheduling algorithm and using the node collaborative scheduling algorithm, the network parameters are the same as the above. Since geographic routing has low latency, this advantage continues to be magnified by combining routing with coverage control techniques.

**Figure 18:**
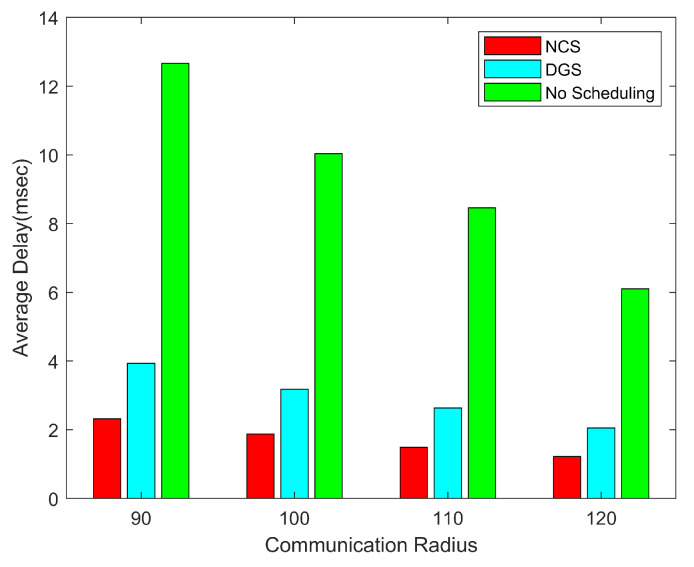
End-to-end average delay with different communication radius.

We can apparently understand from the illustration of Figure 18, the lesser the number of nodes working, the lesser the number of paths generated which results in lesser congestion in the network and lower latency in the network. NCS and DGS reduce the number of nodes working to a great extent and hence the latency is much lower than when coverage scheduling algorithms are not used. As the communication radius increases, the number of hops for data transmission will be less and hence the average delay of the network is lower. NCS has the least number of working nodes which leads to the lowest delay.

**Figure 19:**
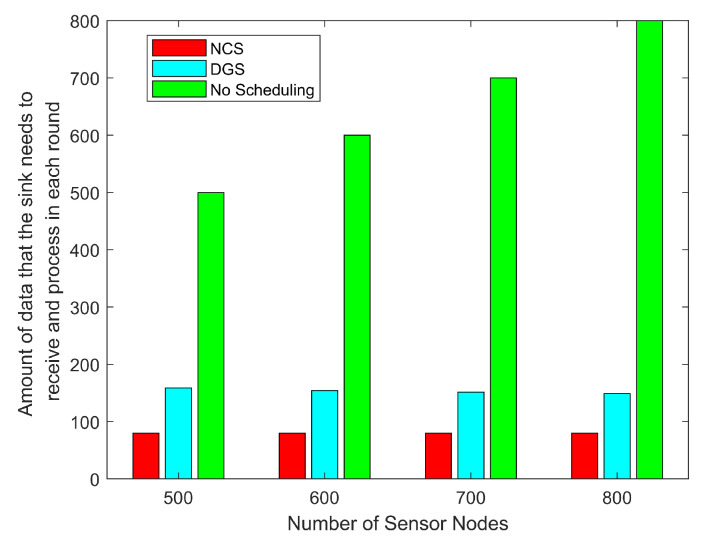
Amount of data that the sink needs to receive and process in each round with different number of nodes.

Finally, we analyze the complexity of the proposed algorithms. As far as the complexity analysis of the algorithms for each sensor node is concerned, they all derive their computation from the information interactions with the neighboring nodes; the location and remaining energy of each neighboring node need to be obtained in the improved geographic routing protocol. A few nodes need to obtain the new location of the sink in the tree-based routing protocol; the same is conducted for obtaining the valid state and remaining energy of the neighboring nodes in the link detection and repair protocol. Each protocol relies on these interactions to take the next action, and only one loop needs to be executed. Therefore, the program running inside each sensor node has an algorithmic complexity of O(n), the size of *n* depends on the number of neighboring nodes. In addition, after the NCS algorithm, the amount of data received by the sink within each round is greatly reduced, which means that the sink does not have to receive and process a large amount of data within a short period of time, which greatly reduces the burden of the sink and contributes to the stability of the whole network. Figure 19 demonstrates this situation, after combining the coverage control algorithms, the network can transmit the least amount of data under the premise of guaranteeing the coverage, reducing the burden of sink and also filtering out the repeated and redundant data, Especially in large-scale sensor networks, combining the routing algorithms with the coverage control technique is necessary, and the NCS adopted in this paper further amplifies this advantage.

Table 5 shows the summary of the critical simulation results in comparison with the baselines. It can be clearly observed that ERG-NCS has an excellent performance in several important performances of WSN routing technology research, which has the best network lifetime, packet delivery rate, throughput, delay and network overhead. ERG-NCS, as a novel routing technology with comprehensive performance, will have great application value in the field of WSN.

## 5. Conclusions

The geographic routing protocol with its simple structure, low energy consumption as well as high scalability has attracted a lot of attention from researchers in recent years; however, its blindness in data transmission has been a major challenge for network reliability. This paper fundamentally solves this problem by link detection and repair algorithm, where each node can judge the validity of its own communication status. In addition, the existing routing protocols always use all the nodes to work, and lack of literature systematically combining routing protocols with coverage control techniques, which is the core work conducted in this paper, in which the proposed node collaborative scheduling algorithm breaks through the limitations of the traditional disk coverage model so that the whole network has a greater improvement in network lifetime, packet delivery rate, network coverage, and delay so that such a comprehensive and robust routing scheme will make a good contribution to unmaintained and unmanaged application scenarios. We hope to continue deepening the research in this field, using scientific and systematic solutions to solve more important problems in WSN.

## Figures and Tables

**Table 1:** Summary of previous works.

Protocol	Method	Advantage	Disadvantage
FC-CRAs [9]	Combining clustering	Low energy consumption	Single hop
D2CRP [10]	Combining clustering	High lifetime	Energy imbalance
ESCVAD [11]	Combining clustering	High lifetime	Single hop
PPO [12] MADRL [13]	Deep learning	High PDR	High overhead
RFLQGeo [14]	Deep learning	Low overhead	Energy imbalance
RR [15] ERSMR [16]	Ring routing	High lifetime	High delay
DGRP [17] HESTOR [18]	Ring routing	Low delay	Energy imbalance
MSGR [19] VGB [20]	Grid structure	Low energy consumption	Low PDR
CESMA-MTRS [21]	Grid structure	Low delay and high PDR	Do not support mobile sink
STTD [22] MCDM [23]	Mobile sink	Energy balance	High latency
EEB [24]	Mobile sink	Energy balance	Low PDR
RTG [25]	Mobile sink	High lifetime and low delay	Low PDR
AMSG [26]	Mobile sink	High lifetime and high throughput	Low PDR
EPSO [27] UDMC [28] OPT [29] GRNN [30]	Coverage study	Fewer nodes used	No routing protocols and no node collaboration

**Table 2:** Simulation parameters.

Parameter	Value
The Network size	1000 m × 1000 m
Number of nodes	500–800
Communication radius	90 m–120 m
Coverage radius	50 m
Data packet size	50 bytes
Control packet size	250 bits
Initial energy	2 J
Coverage rate threshold	0.9
*a*	250 m
λ	0.5
$E_{elec}$	50 nJ/bit
$\varepsilon_{fs}$	10 pJ/bit/m^2^
$\varepsilon_{mp}$	0.0013 pJ/bit/m^4^

**Table 3:** The algorithms’ network PDR with different number of sensor nodes.

Nodes Number	500	600	700	800
ERG-NCS	0.9357	0.9409	0.9573	0.9683
ERG-DGS	0.9172	0.9237	0.9458	0.9508
AMSG-DGS	0.6161	0.6683	0.7100	0.7447
RTG-DGS	0.6308	0.7045	0.7558	0.7801
ERG	0.8501	0.8644	0.8838	0.8968
AMSG	0.5734	0.6281	0.6630	0.6976
RTG	0.6245	0.6677	0.7148	0.7374

The underlined value is the highest PDR for each node number.

**Table 4:** The algorithms’ network lifetime with different numbers of sensor nodes.

Nodes Number	500	600	700	800
ERG-NCS	6449.7	7640.5	8716.3	10,056.5
ERG-DGS	3475.1	4114.6	5188.4	5631.5
AMSG-DGS	5906.4	6662.7	7545.8	8320.6
RTG-DGS	4930.2	5780.2	6446.8	7043.7
ERG	1229.4	1242.9	1235	1259.5
AMSG	2155.6	1949.6	1856	1734.8
RTG	1829.3	1665.3	1561.2	1474

The underlined value is the highest lifetime for each node number.

**Table 5:** The summary of the critical simulation results in comparison with the baselines.

Performance	ERG-NCS	AMSG-DGS	RTG-DGS	AMSG	RTG
Network lifetime	Highest	High	Average	Low	Low
PDR	High	Average	Average	Low	Low
Throughput	Highest	High	Average	High	Average
Delay	Lowest	Low	Low	High	High
Overhead	Low	Average	Average	High	High

## Data Availability

Data are contained within the article.
